# A Key Role in Catalysis and Enzyme Thermostability of a Conserved Helix H5 Motif of Human Glutathione Transferase A1-1

**DOI:** 10.3390/ijms24043700

**Published:** 2023-02-12

**Authors:** Evangelia G. Chronopoulou, Lana Mutabdzija, Nirmal Poudel, Anastassios C. Papageorgiou, Nikolaos E. Labrou

**Affiliations:** 1 Laboratory of Enzyme Technology, Department of Biotechnology, School of Applied Biology and Biotechnology, Agricultural University of Athens, 75 Iera Odos Street, 11855 Athens, Greece; 2 Turku Bioscience Centre, University of Turku and Åbo Akademi University, 20520 Turku, Finland

**Keywords:** alpha class glutathione transferase, site-directed mutagenesis, conserved amino acids, motif-based protein engineering, thermostability

## Abstract

Glutathione transferases (GSTs) are promiscuous enzymes whose main function is the detoxification of electrophilic compounds. These enzymes are characterized by structural modularity that underpins their exploitation as dynamic scaffolds for engineering enzyme variants, with customized catalytic and structural properties. In the present work, multiple sequence alignment of the alpha class GSTs allowed the identification of three conserved residues (E137, K141, and S142) at α-helix 5 (H5). A motif-directed redesign of the human glutathione transferase A1-1 (hGSTA1-1) was performed through site-directed mutagenesis at these sites, creating two single- and two double-point mutants (E137H, K141H, K141H/S142H, and E137H/K141H). The results showed that all the enzyme variants displayed enhanced catalytic activity compared to the wild-type enzyme hGSTA1-1, while the double mutant hGSTA1-K141H/S142H also showed improved thermal stability. X-ray crystallographic analysis revealed the molecular basis of the effects of double mutations on enzyme stability and catalysis. The biochemical and structural analysis presented here will contribute to a deeper understanding of the structure and function of alpha class GSTs.

## 1. Introduction

Glutathione transferases (GSTs) are promiscuous enzymes that are widely distributed in both eukaryotes and prokaryotes, contributing significantly to the detoxification, metabolism and transport or sequestration of endogenous and xenobiotic compounds [1,2,3]. The eukaryotic cytosolic GST family is composed of different classes based on chemical, physical, and structural properties. In humans, seven classes have already been characterized: alpha, zeta, theta, mu, pi, sigma, and omega [2]. Alpha, mu, pi, and sigma classes compose the Tyr-GST group, since the Tyr residue interacts with and activates the GSH substrate [1,2]. The human alpha class of GSTs (hGSTAs) contains five isoenzymes (hGSTA1-1−hGSTA5-5), comprising one of the most evolved GST classes. Although predominantly found in the cytosol, these enzymes seem to play an essential role in cell physiology, since they can also be found in the “nuclear shield”, providing DNA protection [4,5]. The isoenzyme hGSTA1-1 is primarily expressed in the human liver but can also be found in various other human tissues [6]. It is a key enzyme in human physiology, since it is involved in the phase II detoxification mechanism of a wide range of electrophilic compounds, such as drugs, carcinogenic metabolites, and environmental pollutants [7,8,9].

GSTs’ facile expression in recombinant *E. coli* system purification and stability, along with their catalytic and binding features, are significant advantages that reinforce their exploitation in several areas of biotechnology [10,11,12,13]. The redesign of enzymes through protein engineering is a powerful strategy to manipulate a structure and reveal structure–function and structure–stability relationships [14,15,16]. Therefore, the engineering of GSTs can lead to the construction of new variants with desired catalytic and structural properties that are appropriate for biotech applications [16,17], such as biosensor development or even the creation of protein nanostructures [12,13]. 

Cytosolic GSTs are dimeric enzymes with each subunit having two distinct domains: a conserved N-terminal thioredoxin-like α/β domain and a C-terminal α-helical domain [2,18]. The C-terminal domain (domain 2, residues 85–207) of hGSTA1-1 consists of helices H4-H9 [19]. GSTs are characterized by at least two ligand-binding sites: the G-site, which is responsible for GSH-binding, and the H-site, which binds different electrophilic substrates [2,20,21]. It is well-known that the main contributors in hGSTA1-1 catalysis are the upper parts of the C-terminal helices H4 and H9 of the protein that acts as a lid over the active site [22]. In addition, the hydrophobic staple motif that corresponds between residues Leu153 and Ile158 is highly conserved to GSTs. The residues of this motif participate in interdomain interactions and have been found to be crucial for hGSTA1-1 stability and GST rapid folding [14].

In the present study, new “hot-spot” residues (E137, K141, and S142) were selected by in silico analysis and assessed by site-directed mutagenesis. The amino acids that were studied belong to a conserved motif of hGSTA1-1 α-helix H5. Two single- and two double-point mutants (E137H, K141H, K141H/S142H, and E137H/K141H) were created for investigation. To the best of our knowledge, the amino acids E137 and K141 have not been studied before, while S142 has been previously reported by Nicolaï et al. [23] as one of the residues involved in ligand binding. Furthermore, for gaining a deeper insight into the role of these residues, the crystal structure of hGSTA1-1-K141H/S142H was determined by X-ray crystallography.

## 2. Results and Discussion

### 2.1. Selection of Conserved “Hot-Spot” Residues of Alpha-Class GSTs and Prediction of Their Impact on hGSTA1-1 Flexibility

**Figure 1:**
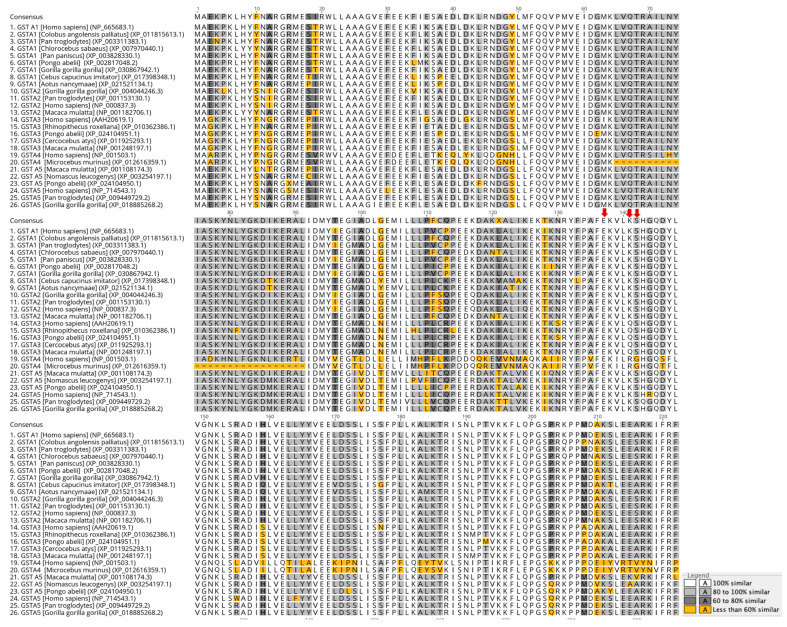
Multiple amino acid sequence alignment of alpha class GSTs colored according to similarity. Red arrows depict the amino acids of the helix H5 motif that were selected in the present study. The figure was created using the Geneious v9.1.2 [29].

Amino acid sequence alignment of alpha class GST members from different mammals was carried out (Figure 1). The results showed that the amino acids E137, K141, and S142 belong to the conserved motif FΕKVLKS (region 136–142) of H5 helix, and they are not directly involved in the formation of the substrate-binding sites of hGSTA1-1. The selected amino acids have not been studied before; however, S142 has been reported as one of the amino acids, along with H143, that take part in the network that contributes to ligand binding of hGSTA1-1 [23]. In addition, the F136 site of the mu class GST from the helminth parasite *Fasciola gigantica* was found to be significant for the catalytic function and rigidity of the enzyme [18]. This study included the substitution of residues K141 and S142 with histidine by site-directed mutagenesis. In particular, histidine was selected as the replacement residue because of the significant role of histidine in catalysis, protein–protein interactions [12,24], and metal binding [25]. These properties of histidine could endow hGSTA1-1 with features that can make it a promising scaffold for future applications in nanobiotechnology or in the development of artificial metalloenzymes [26]. Moreover, histidine does not alter the hydrophilic feature of the motif, which probably could cause destabilization effects on the enzyme structure. Considering all the above, a computational study and protein engineering experiments for the mutation of the amino acids at the sites 137, 141, and 142 to histidine (E137H, K141H, and S142H) were undertaken.

**Figure 2:**
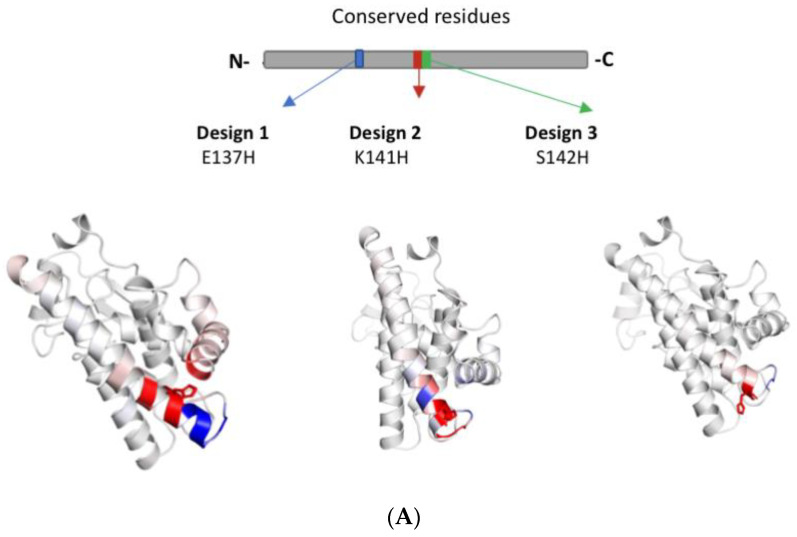

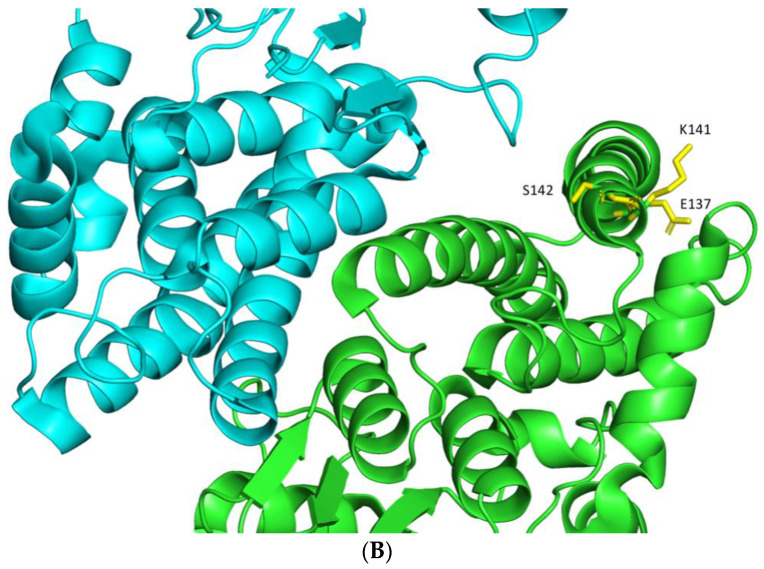
Computational assessment of hGSTA1-1 flexibility by using DynaMut [27]. (**A**) Amino acids are colored according to the vibrational entropy change upon mutation. The blue color represents a rigidification of the structure, and the red one a gain in flexibility. (**B**) The positions of the selected residues are shown in the native structure hGSTA1-1 (PDB code: 1PKZ) as sticks of yellow color. The different chains of the dimer are depicted in cyan and green color. The figure was created using the PyMOL Molecular Graphics System [30].

The effect of the mutations on hGSTA1-1 flexibility was first assessed using the DynaMut software [27] and the structure of hGSTA1-1 (PDB code 1PKZ) [28]. The results showed that the mutations E137H, K141H, and S142H can affect enzyme flexibility (Figure 2A). Taking into consideration this difference, we proceeded to create single and double mutations on these sites (Figure 2B), and four mutants were created: hGSTA1-E137H, hGSTA1-K141H, hGSTA1-K141H/S142H, and hGSTA1- E137H/K141H.

### 2.2. Steady-State Kinetics Analysis

The wild-type hGSTA1-1 enzyme and its variants were expressed in *E. coli* BL21 (DE3) cells and purified (70–100% recovery of activity) by affinity chromatography on an immobilized GSH column. SDS-PAGE analysis suggested that the wild-type and the mutant enzymes were suitably purified for kinetics and structural analysis (Supplementary Figure S1).

**Figure 3:**
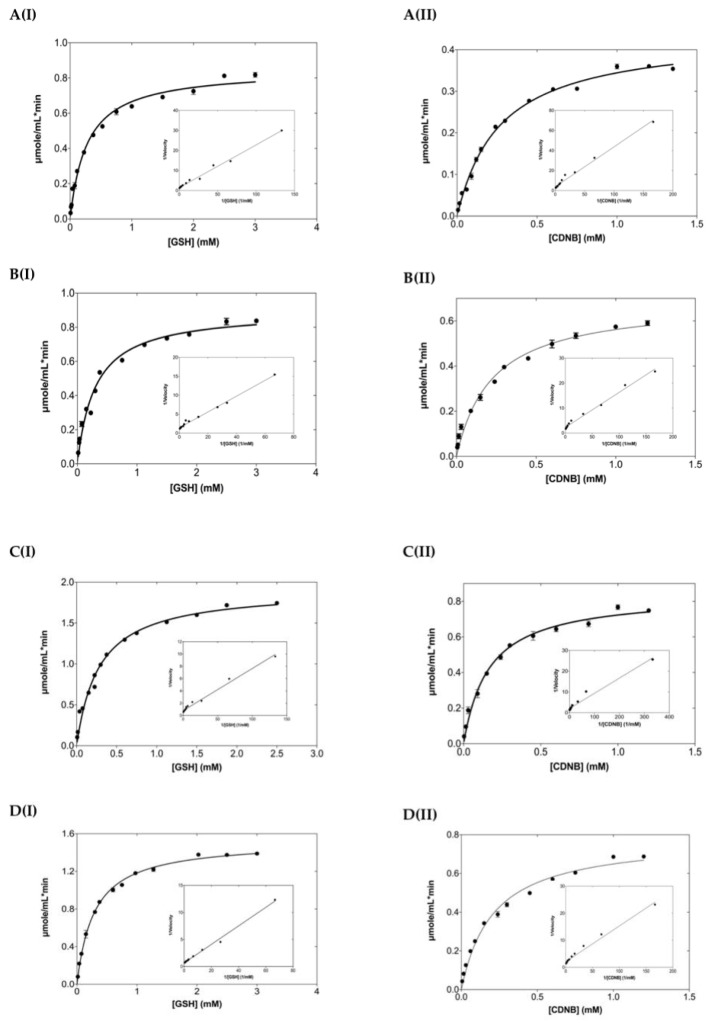

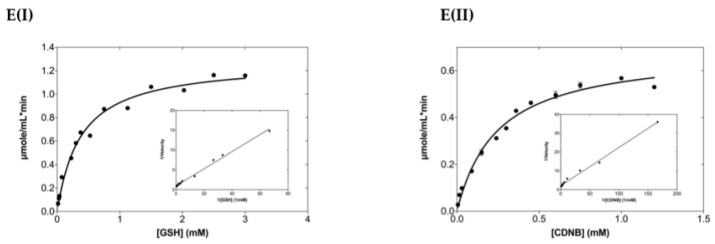
Steady-state kinetic analysis of hGSTA1-1 and its mutants using GSH as a variable substrate (I) and CDNB at a fixed concentration. Steady-state kinetic analysis of hGSTA1-1 and its mutants using the CDNB as a variable substrate (II) and GSH at a fixed concentration. (**A**) hGSTA1-1, (**B**) hGSTA1-K141H, (**C**) hGSTA1-K141H/S142H, (**D**) hGSTA1-E137H, and (**E**) hGSTA1-E137H/K141H. Experiments were performed in triplicate. The inside graphs are the Lineweaver-Burk plots.

Steady-state kinetics analysis (Figure 3) was performed using as substrate the GSH/CDNB model reaction system, a typical SN2 nucleophilic substitution reaction. The kinetic parameters are listed in Table 1. The results showed that all the mutants obeyed normal Michaelis-Menten kinetics for both substrates. The kinetic parameters of the enzyme for GSH and especially for CDNB were significantly affected by the mutations. The K_m_ and k_cat_/K_m_ values for CDNB were decreased and increased, respectively. Significant improvement of catalytic efficiency for CDNB (approximately 1.7–3.3 folds) was observed following the descending order of enhancement: hGSTA1-K141H/S142H (40% reduction of K_m_) > hGSTA1-E137H (22% reduction of K_m_) > hGSTA1-K141H (17% reduction of K_m_) > hGSTA1-E137H/K141H (8% reduction of K_m_). The lower K_m_ values for CDNB suggest that the mutations in H5 provoke changes in the structure of the H-site. However, the effect of mutations on the K_m_ for GSH was subtle. All the enzyme mutants exhibited enhanced catalytic efficiency, in particular the mutants hGSTA1-K141H/S142H and hGSTA1-E137H. The contribution of each mutation on the difference free energy change (ΔΔG) (Equation (1)) for stabilization of the transition state (ΔΔG wt → mutant) [31] is shown in Table 2. The results demonstrate that the selected motif causes a significant effect on catalysis. The catalytic efficiency k_cat_/K_m_ was higher for all the studied mutants compared to wild-type hGSTA1-1, indicating that the selected residues are important for the stabilizing effect and the reduction of the free energy of the transition state. Therefore, the mutated sites of this study (E137, K141, and S142) along with the F220 and F222 residues seem to influence the rate-determining step of the catalytic reaction [15].

### 2.3. The Dependence of Enzyme Activity on pH

**Figure 4:**
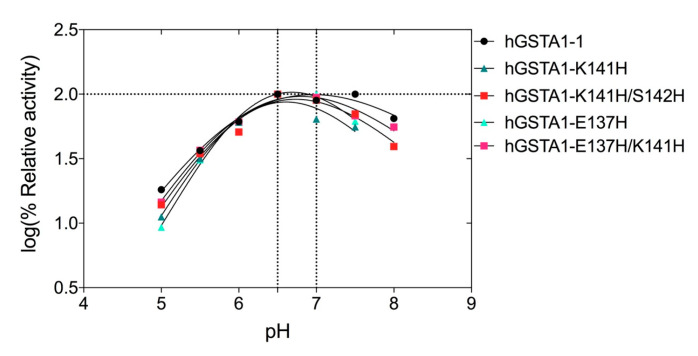
Effect of pH on enzyme activity for hGSTA1-1 and its mutants. Enzyme activity was measured in the standard assay reaction at 37 °C, at pH values ranging from 4 to 9, and using the appropriate buffers.

The dependence of the enzyme activity on pH for the wild-type and the mutant enzymes was assessed over the pH range 4–9, and the results are shown in Figure 4. The results indicate that the mutations do not significantly affect the optimal pH activity of hGSTA1-1, which is in the range of 6.6–6.9 at 37 °C (Figure 4). Below pH 6 and above pH 7, the enzyme activity is strongly diminished.

### 2.4. Thermal Stability of the Wild-Type and Mutant Enzymes

**Figure 5:**
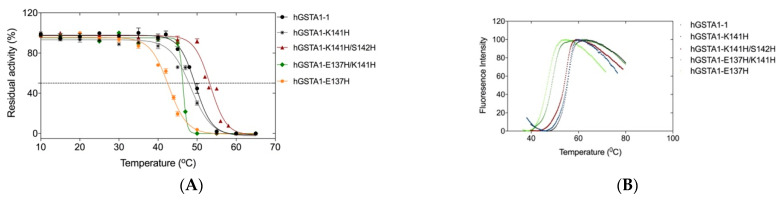
(**A**) Enzyme residual activity curves for hGSTA1-1 (black line), hGSTA1-K141H (grey line), hGSTA1-K141H/S142H (red line), hGSTA1-E137H/K141H (green line), and hGSTA1-E137H (orange line). The residual activities were measured after heat treatment at various temperatures (°C) for 5 min. Values presented are given as a mean of triplicates ± SE. (**B**) Melting curve measurements by DSF (scan rate of 1 °C/min) for hGSTA1-1(black), hGSTA1-K141H (blue), hGSTA1-K141H/S142H (red), hGSTA1-E137H/K141H (green), and hGSTA1-E137H (light green).

To assess the effect of the mutations on the enzyme’s structural stability, thermal inactivation kinetics and fluorescence unfolding measurements using differential scanning fluorimetry (DSF) were performed (Figure 5). The kinetics of thermal inactivation were measured in the range of 10–65 °C (Figure 5A). The thermal half-inactivation temperature T_1/2_ for the wild-type and the mutant enzymes is listed in Table 3. The results indicated that the mutant hGSTA1-K141H exhibits almost identical T_1/2_ to that of the wild-type enzyme. In contrast, hGSTA1-K141H/S142H depicted an increase in the T_1/2_ value by approximately 4 °C (T_1/2_ 53.4 ± 0.2 °C), suggesting that the double mutant hGSTA1-K141H/S142H displayed higher thermal stability.

DSF is widely used as a method to estimate both the folding state and thermal stability of proteins [32]. The temperature-induced unfolding profiles of the wild-type and mutants were studied by measuring the corresponding changes in fluorescence emission upon temperature increase (Figure 5B). The unfolding profiles of the enzymes exhibited a single peak with maximum fluorescence intensity, corresponding to T_m_ values. The results showed that the mutants hGSTA1-E137H and hGSTA1-K137H/S141H display lower T_m_ values, whereas the T_m_ values for hGSTA1-K141H and hGSTA1-K141H/S142H are almost identical to that determined for the wild-type enzyme (Table 4).

Inspection of the entire data set of T_m_ and T_1/2_ revealed that the T_m_ values lie between 46.2 and 55.0 °C, slightly larger than the T_1/2_ values, which range between 42.7 and 53.4 °C. The observed differences are presumably the consequence of the different parameters measured (loss of enzyme activity vs. fluorescence structural unfolding), suggesting that these two features are possibly governed by different mechanisms. 

The thermostability of the enzymes was assessed through the standard Gibbs free energy of unfolding (ΔuG°) while taking into consideration the T_m_ [33] (Table 4). For the estimation of the standard free energy of stabilization, the unfolding process was considered as a two-state process. The results showed changes between the wild-type and the mutants. The wild-type enzyme exhibits a T_m_ of 55.0 °C, which would initially suggest that it is more stable than the hGSTA1-K141H/S142H mutant, which exhibits a T_m_ of 53.2 °C; however, under standard conditions, the ΔuG° of the mutant hGSTA1-K141H/S142H (52.1 kJ mol^−1^) is 0.4 kJ mol^−1^ higher than the ΔuG° of the wild-type (51.7 kJ mol^−1^). The mutant hGSTA1-K141H, though it exhibits a similar T_m_ (54.5 °C) to the wild-type enzyme, has a significantly lower Gibbs free energy of unfolding under standard conditions (ΔuG° 29.3 kJ mol^−1^). These results point to the conclusion that the double mutant hGSTA1-K141H/S142H displays higher stability compared to the wild-type enzyme and the K141H single mutant.

### 2.5. Probing the Ligandin-Binding Properties of hGSTA1-1 and Its Mutants with Pesticides

**Figure 6:**
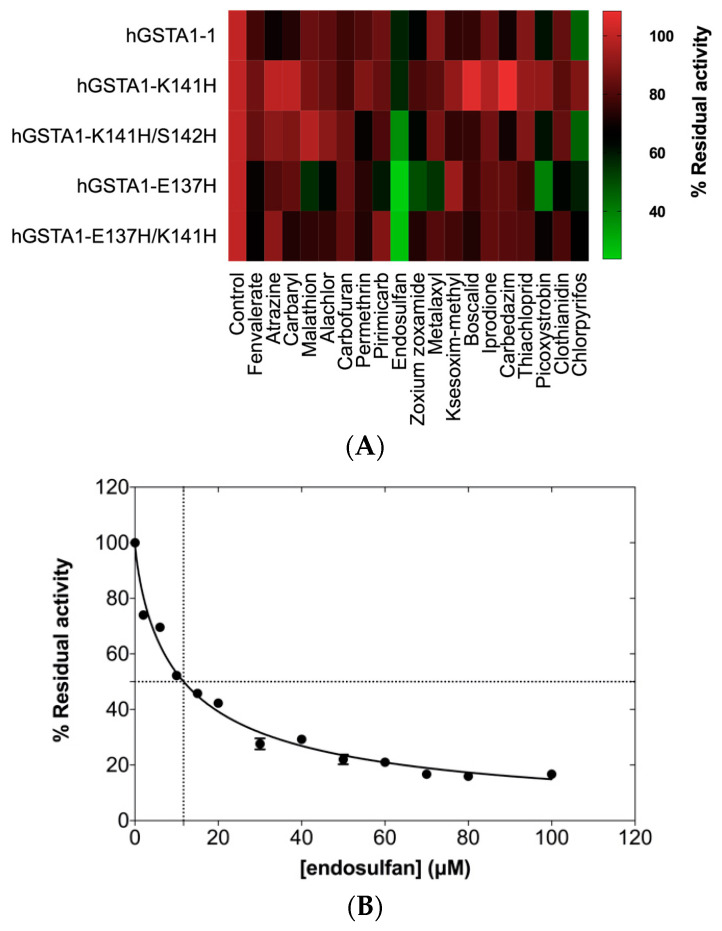
(**A**) Screening of inhibition potency of selected pesticides (25 μΜ) against the wild-type enzyme and its mutants. The inhibition was evaluated by measuring GST catalytic activity using GSH-CDNB as substrate system, in the presence of 25 μΜ of pesticide for a period of 2 min. In the absence of pesticides, enzyme activity was taken as 100%. Experiments were performed in triplicate, and the reported values in the heat map correspond to the mean value. (**B**) Concentration–response curve for endosulfan. IC50 value for hGSTA1-K141H/S142H was determined using GraphPad Prism. GST activity was assayed using the CDNB–GSH assay system in triplicate (standard errors are depicted).

The enzyme hGSTA1-1 exhibits well-defined ligand-binding properties and can bind a range of xenobiotics (e.g., pesticides) in a non-substrate manner [34]. Binding of xenobiotics to hGSTA1-1 leads to the inhibition of the enzyme’s activity [9]. To assess the effect of mutations at positions 137, 141, and 142 on the enzyme’s ligandin function, screening against various pesticides was carried out (Figure 6). As shown in Figure 6A, the wild-type and the mutant enzymes are inhibited by several pesticides. The mutants hGSTA1-K141H/S142H, hGSTA1-E137H/K141H, and hGSTA1-E137H exhibit higher sensitivity towards endosulfan compared to the wild-type enzyme, whose activity is inhibited >50% by the organophosphate insecticide chlorpyrifos. The results suggest that the amino acids at positions 137, 141, and 142 seem to play a significant role in the ligand-binding properties of hGSTA1-1. A concentration-response curve for the hGSTA1-K141H/S142H mutant is illustrated in Figure 6B, and the IC50 value was found be equal to 11.64 ± 0.50 μΜ.

### 2.6. Crystallographic Analysis and Structural Characterization of hGSTA1-K141H/S142H

**Figure 7:**
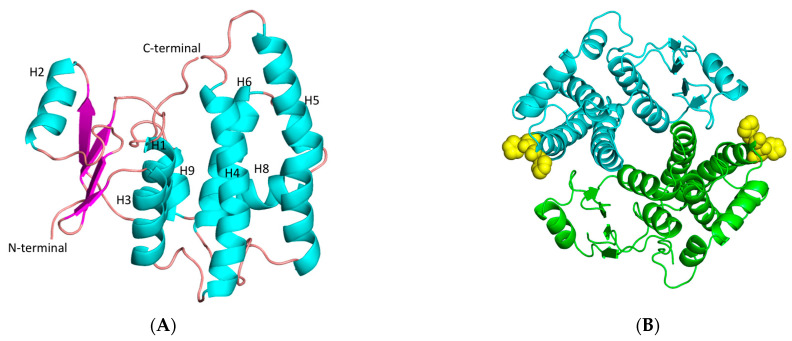
(**A**) Representation of hGSTA1-K141H/S142H subunit. Helices and strands are shown in cyan and magenta, respectively. The helices are labeled. (**B**) Representation of hGSTA1-K141H/S142H dimer from the top. The two-fold axis that relates the two molecules is perpendicular to the plane of the paper. The histidine amino acids at positions 141 and 142 are shown as yellow spheres. The figures were created using PyMOL Molecular Graphics System [30].

The improvement in the catalytic activity of hGSTA1-K141H/S142H as well as its thermal stability prompt us to investigate the structure of this mutant at the atomic level using X-ray crystallography (Figure 7). The mutant was crystallized in two crystal forms with four molecules (1.56 Å resolution) and two molecules (1.87 Å), respectively, in the crystallographic asymmetric unit. Both forms display the typical dimer state found in other GSTs [35,36]. The final structure in both crystal forms (Table 5) exhibits good geometry, as judged by several quality criteria. Residues 212–221 at the C-terminal were not included in the structure because of high disorder. The high-resolution structure has Cys112 modified to S,S-(2-hydroxyethyl)thiocysteine, owing to longer storage of the enzyme prior to crystallization. Apart from this, the two structures have only subtle differences, with a root mean square deviation (rmsd) of 0.43 Å.

**Figure 8:**
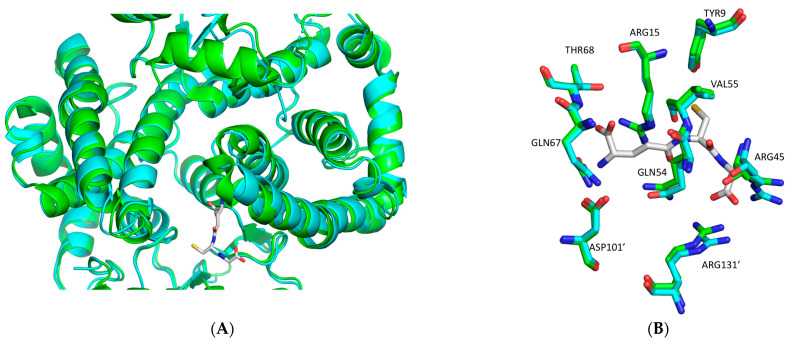
(**A**) Structural superposition of hGSTA1-K141H/S142H (cyan) onto hGSTA1-1−GSH (green) (PDB code: 1PKW) [28]. GSH is shown in stick representation. (**B**) Close-up comparison of hGSTA1-K141H/S142H and hGSTA1-1 (PDB code: 1PKW) active sites after superposition. The residues that are involved in GSH binding are Tyr9, Arg15, Arg45, Glu54, Val55, Gln67, and Thr68, and Asp101′ and Arg131′ from the second subunit. The structures of hGSTA1-K141H/S142H and hGSTA1-1− GSH are colored in cyan and green, respectively. The analysis was carried out using PyMOL Molecular Graphics System [30].

The analysis revealed that each monomer of hGSTA1-K141H/S142H retains the well-established structural features of GSTs, which consist of the presence of two distinct domains. At the N-terminal region, a small α/β thioredoxin-like domain with βαβαββα folding topology is formed. The topology is arranged in the order β1, β2, β3, and β4. At the C-terminal region, a large helical domain is formed (Figure 7A). The engineered amino acids are at the surface of the enzyme (Figure 7B). At the end of helix H3, a short linker (residues 79–85) joins the N- and C-terminal domains. The amino acids that form the G-site are illustrated in Figure 8.

**Figure 9:**
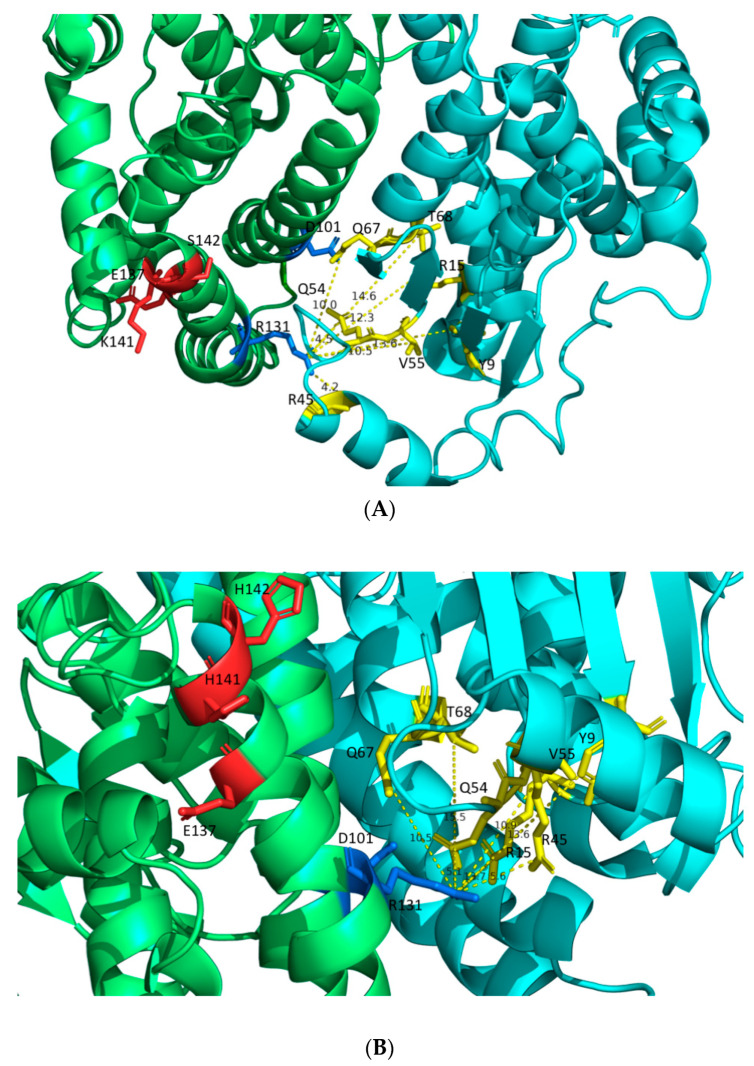
(**A**) Distances between R131′ and G-site residues of hGSTA1 (PDB code: 1PKZ [28]). (**B**) Distances between R131′ and G-site residues of hGSTA1-K141H/S142H. The distances are shown in dashed yellow lines. The G-site residues of the enzymes (Y9, R15, R45, Q54, V55, Q67, and T68) are presented in yellow. The two molecules of the dimer are shown in lime green and cyan. The residues D101 and R131 of the second molecule in the dimer are presented in marine. The residues E137, K141, S142, H141, and H142 are presented in red. The analysis was carried out using the PyMOL Molecular Graphics System [30].

The distances of R131’ from the amino acids of the G-site (Y9, R15, R45, Q54, V55, Q67, and T68) (Figure 9) of hGSTA1-1 and hGSTA1-K141H/S142H are illustrated in Figure 9A,B, respectively. The residue R131 has been reported to play a significant role in the flexibility of the H5 region [23]. Nilsson et al. 2002 concluded that product release is rate-limiting in the steady-state kinetics of CDNB reaction [15]. Therefore, the subtle structural changes and the higher flexibility of the H5 region probably form a more open active site that facilitates the product release.

### 2.7. Residue Interaction Network (RIN) in Selected Motif

The RIN method [37] was used in order to identify important interactions among selected residues in hGSTA1-1 and hGSTA1-1K141H/S142H that interfere with the mutated amino acids. E137 seems to be of particular importance and appears to play a central role in the interaction network, as it is characterized by node degree 9 (i.e., the number of edges that are connected to the node), indicating that it participates in a range of different networks. Therefore, its replacement by His probably induces conformational changes that affect enzyme catalysis and stability. 

**Figure 10:**
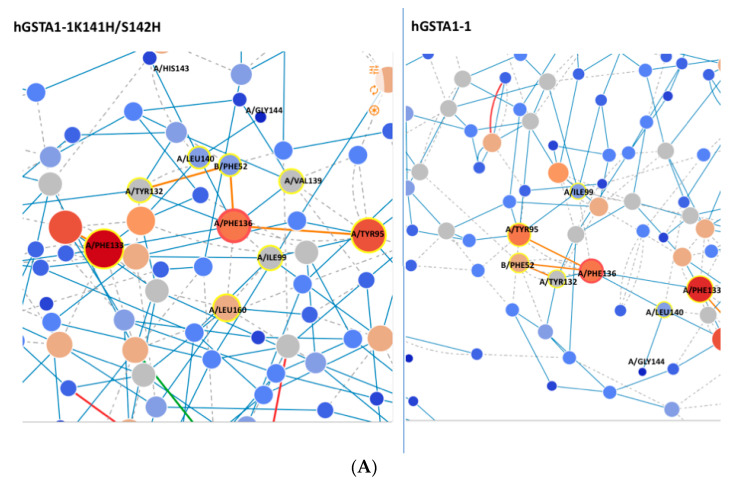

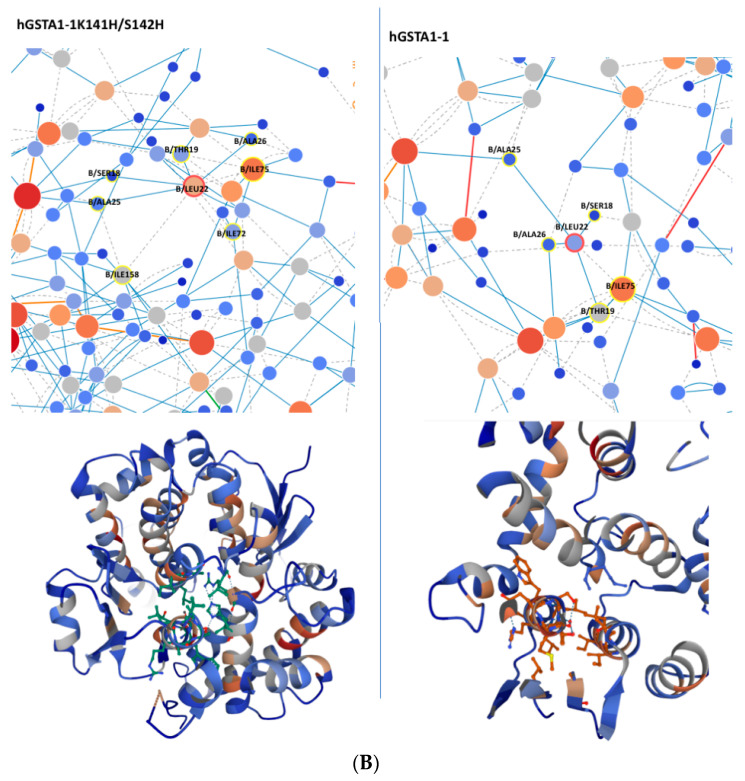
(**A**) Network around Phe136 of hGSTA1-K141H/S142H and hGSTA1-1; (**B**) network around Leu22 of hGSTA1-1. The picture was created with the program RING [37]. Blue, grey, green, and red lines represent H-bond, van de Waals, π-cation, and π-π stack interactions, respectively.

It is well established that the catalysis of hGSTA1-1 is significantly affected by amino acids that contribute to intersubunit communication [38]. It is noteworthy that the amino acids F136 and V139 of the selected motif have been reported to contribute to intersubunit structural communication [38]. Analysis of the network of interactions in the enzymes hGSTA1-1 and hGSTA1-1K141H/S142H with the RING [37] and Cytoscape program [39] showed that the F136 of the mutant interacts with the amino acids V139 and L160. The amino acid L160 has been found to be implicated in ligand binding [23]. In addition, regarding the amino acid Phe52, two van de Waals interactions are missing in the mutant, compared to the wild-type enzyme (Figure 10A). Phe52 forms the intersubunit lock-and-key motif and plays a significant role in the catalytic function and stability of the GST dimer [40]. Therefore, these interactions can be further evidence for mutant stability.

Another amino acid that is likely essential for the enhanced catalytic activity of the hGSTA1-1K141H/S142H is L22, which seems to form van de Waals interaction with I158 (Figure 10B) from helix H6. However, the same interaction is absent in the wild-type enzyme. I158 is considered an important structural element of the hydrophobic staple motif (L153 to I158) because of its hydrophobic interactions with the H1-helix (W21, L22, and A25), which are critical for the stability of hGSTA1-1 [14]. In addition, a difference in node degree for L22 from 5.00 to 7.00 can be seen in Figure 10B, suggesting that L22 of hGSTA1-K141H/S142H is involved in a more extended network of interactions in this mutant. The hydrophobic staple motif has a significant role in the folding of hGSTA1-1 and hGSTP1-1 [14,41]. According to our results, the increased number of interactions observed for L22 probably affects the structure of the hydrophobic staple motif.

## 3. Materials and Methods

### 3.1. Materials

KAPA high fidelity DNA polymerase was purchased from KAPA Biosystems (Wilmington, MA, USA). Plasmid purification kit was obtained from Macherey–Nagel (Düren, Nordrhein-Westfalen, Germany). The pesticides that were used were purchased from Riedel de Haen (Seelze, Germany). GST enzyme substrates and antibiotics were purchased from Sigma-Aldrich, (St. Louis, MO, USA). The In-fusion^®^HD Cloning kit was obtained from Takara Bio, Inc. (Kusatsu, Japan).

### 3.2. Methods

#### 3.2.1. In Silico Analysis

The identification of conserved amino acid “hot spots” in alpha class GSTs was carried out by multiple amino acid alignment of a set of sequences retrieved from a pBLAST search. The alignment was produced by Clustal W [42] and edited in Geneious 9.1.2 software [29]. The impact of the amino acid “hot spots” on protein flexibility was determined using DynaMut software [27]. In silico analysis was carried out using the structure of hGSTA1-1 (PDB code: 1PKZ) [28]. 

In addition, a residue interaction network (RIN) was generated using the RING3.0 web server https://ring.biocomputingup.it/submit/ (accessed on 1 September 2022) [37]. The RING3.0 creates residue interaction networks where the amino acids are represented as nodes and the interactions are in the form of edges. The default generation options were used (closest nodes and multiple edges).

#### 3.2.2. Site-Directed Mutagenesis 

The expression construct pOXO4-hGSTA1-1 [9] was used as a template for site-saturation mutagenesis at positions 141 and 142 using the quick-change PCR method [43]. For the mutation at position 137, overlap PCR was used [44]. Site-saturation mutants were produced using a set of synthetic oligonucleotides, in which the mutation sites 141 and 142 were replaced by a specific codon CAT, coding for the amino acid histidine. In the case of the hGSTA1-1 mutant at the amino acid position 137, primers were designed to perform overlap PCR and directional cloning with In-Fusion HD Cloning Plus (TaKaRa Bio Inc., Kusatsu, Japan), using the pEXP5-CT TOPO vector. The pairs of mutagenic oligonucleotide primers that were used in the PCR reactions are listed in Supplementary Table S1. The PCR contained 7.5 pmol of each primer, 5x Kapa High Fidelity DNA polymerase’s buffer, 300 μΜ of each dNTP, 5 ng of hGSTA1-1 plasmid DNA, and 1 U Kapa HiFi DNA polymerase. The PCR comprised 30 cycles of denaturation at 98 °C for 20 s, annealing at different temperatures for 15 s, and polymerization at 72 °C for 1 min. A final extension time at 72 °C for 10 min was performed, after the 30th cycle. The final product was subjected to DpnI digestion. The recombinant plasmids hGSTA1-E137H and hGSTA1-E137H/K141H were constructed following the instructions for the In-fusion^®^HD Cloning kit (TAKARA) using the pEXP5CT-TOPO/TA vector. The mutated plasmids DNA (pOXO4 and pEXP5-CT/TOPO/TA vectors) were used to transform competent *E. coli* DH5a and Stellar cells, respectively. Transformed cells were selected on LB agar plate containing chloramphenicol (33 μg/μL) and ampicillin (100 μg/mL) for the plasmids pOXO4 and pEXP5-CT/TOPO/TA, respectively.

#### 3.2.3. Heterologous Expression and Purification of the Wild-Type and Mutant Enzymes

Recombinant *E. coli* cells BL21(DE3) with the wild-type and mutant constructs, were grown at 37 °C in Luria-Bertani (LB) medium containing ampicillin (100 μg/mL) or chloramphenicol (33 μg/mL). Protein expression and enzyme purification were performed as described by Chronopoulou et al. [9]. SDS PAGE analysis was used for the estimation of protein purity [45]. Before biochemical characterization and analysis, the purified enzymes were subjected to extensive dialysis against potassium phosphate buffer (20 mM, pH 7).

#### 3.2.4. Kinetic Analysis of hGSTA1-1 and Its Mutants

GST assays were performed by measuring the conjugation reaction between CDNB (1 mM) and GSH (2.5 mM) at 340 nm (ε = 9.6 mM^−1^·cm^−1^), 37 °C, and pH 6.5, as previously described [28]. Steady-state kinetic analysis for the kinetic parameters’ determination was carried out at pH 6.5 and 37 °C using GSH and CDNB as substrates. Reaction rates were corrected for non-enzymic rates. For the determination of K_m_ for GSH, the concentration of CDNB was kept at 1 mM, while GSH was varied (0.015–3 mM). For the determination of K_m_ for CDNB, the concentration of GSH was kept at 2.5 mM, and the concentration of CDNB was varied (0.006–1.350 mM). Typical measurements for the steady-state kinetics plot were carried out in triplicate, with maximum deviations from the mean value of 10%. Curve-fits were obtained using the GraphPad (GraphPad Software Inc., San Diego, CA, USA, Version 7.00) computer program.

The contribution of each mutation in the difference free energy change (ΔG) for stabilization of the transition state (ΔΔG) was calculated using the following equation [31]:

(1) $$\Delta \Delta G=-RTln\left(\frac{\frac{kcat}{Km}\;of\;mutant}{\frac{kcat}{Km}\;of\;wild\;type}\right)$$

#### 3.2.5. pH Dependence of Kinetic Parameters

For the record of pH profile and the determination of the enzymes’ optimal pH, enzyme assays were carried out at 37 °C in 0.1 M sodium acetate buffer (pH 4.0–6.0), 0.1 M potassium phosphate buffer (pH 6.0–7.0), and 0.1 M Trizma base buffer (pH 7.0–9.0). All measurements were corrected for the non-enzymic reaction using control reactions, where no enzyme was added.

#### 3.2.6. Thermal Stability

The thermal stability of the wild-type and mutant enzymes was investigated using kinetics inactivation studies [46] and differential scanning fluorimetry (DSF) [47]. Kinetics inactivation studies were performed through the determination of residual enzyme activity in the range of 10–65 °C (5 min incubation). Measurements of residual enzyme activity were conducted to determine the temperature at which 50% of the initial enzyme activity is lost after heat treatment (T_1/2_) and were defined from the plot of relative inactivation (%) versus temperature (°C).

DSF was performed on a Real-time PCR StepOneTM instrument (Applied Biosystems, Waltham, MA, USA). The thermal stability was measured in potassium phosphate buffer (20 mM, pH 7) using SYPRO orange dye. Fluorescence monitoring was carried out at 10–95 °C with a ramping rate of 1%. The sigmoidal curves that were generated from the plots of fluorescence intensity versus temperature allowed the determination of the inflection point of the curve (corresponds to T_m_) using the Boltzmann Equation (2) implemented in the GraphPad (GraphPad Software Inc., San Diego, CA, USA, Version 7.00) computer program:

(2) $$y=LL+\frac{UL-LL}{1+\exp\left(Tm-\frac{x}{a}\right)}$$

#### 3.2.7. ΔΔG Calculations from T_m_

The determination of ΔG of enzyme unfolding was measured using fluorescence studies [33]. Fluorescence intensities were used to calculate the fraction of the folded (P_f_) and unfolded (P_u_) state of the enzymes at different temperatures, from the minimum fluorescence (*F_min_*) to the maximum fluorescence (*F_max_*), following the Equation (3).

(3) $$P_{f}=1-\frac{F-F_{min}}{F_{max}-F_{min}}$$

The standard Gibbs free energy of protein unfolding, ΔuG°, is reported as the value of ΔuG° extrapolated back to 298K using the line of best fit from the plot of ΔuG° versus temperature (Supplementary Figure S2) [33].

#### 3.2.8. Inhibition Analysis and Screening of the Wild-Type and hGSTA1-1 Mutants

GST inhibition analysis was performed using the CDNB/GSH system, in the presence or absence of 25 μM pesticide diluted in acetone (fenvalerate, atrazine, carbaryl, malathion, alachlor, carbofuran, permethrin, pirimicarb, endosulfan, zoxium zoxamide, metalaxyl, ksesoxim-methyl, boscalid, iprodione, carbedazim, thiachloprid, picoxystrobin, clothianidin, chlorpyrifos). During the course of the assay (30–60 s), no measurable pesticide/GSH conjugation was observed. The IC50 values were determined by fitting the concentration-response data to Equation (4):

(4) $$\%\;\mathrm{inhibition}=\frac{100}{1+\left(\frac{IC50}{\left[I\right]}\right)}$$

where [I] is the pesticide concentration. The IC50 values were determined using the program GraphPad Prism version 7.00.

#### 3.2.9. Protein Determination

Protein concentration was determined by the Bradford assay, using BSA (fraction V) as a standard protein [48].

#### 3.2.10. Crystallization and Data Analysis

The protein (10 mg/ml in 10 mM Hepes-NaOH, 150 mM NaCl, NaN_3_ 0.002% *w*/*v*, pH 7.0) was crystallized at 16 °C in 24-well Linbro plates using 2 μl of protein solution mixed with an equal volume of precipitant solution. Two conditions were identified to produce good quality crystals. X-ray diffraction data to 1.87 Å from crystals grown in PEG 4K (17–20% *w*/*v*) Na malonate 0.2 M were collected on station P14 at EMBL-Hamburg using an EIGER detector. The space group was *P*2_1_2_1_2_1_. X-ray diffraction data from crystals produced with 20% *w*/*v* PEG 3350, 0.2 lithium citrate tribasic tetrahydrate were collected on EMBL-Hamburg P13 beamline, using a PILATUS 6M detector. These crystals diffracted to 1.56 Å, and the space group was *P*2_1_. In both cases, the crystals were flash-frozen in liquid nitrogen using 20% *v*/*v* glycerol as a cryoprotectant, and the data collection took place at 100 K.

#### 3.2.11. Structure Determination, Refinement, and Analysis

The crystal structures were determined by molecular replacement using the wild-type hGSTA1-1 crystal structure as the search model (PDB code:1GSE) [49]. The structures were refined with Phenix 1.20 [50]. Inspection of the structures and rebuilding was carried out using Coot [51]. Validation tools in Coot and Phenix were used to assess and improve the quality of the structures. X-ray data collection, processing, and refinement statistics are given in Table 5.

## 4. Conclusions

In this study, insights are provided regarding the effects of point mutations E137H, K141H, and S142H of helix H5 on the catalytic activity, xenobiotic selectivity, and structural stability of hGSTA1-1. The results showed that the mutant hGSTA1-K141H/S142H exhibits higher catalytic efficiency, thermal stability, and sensitivity against α-endosulfan compared to the wild-type enzyme. The double mutation K141H/S142H, according to the crystallographic analysis, results in subtle but specific changes in the protein structure, and the observed stability may be attributed to specific interactions within an amino acid network. The increase of flexibility that these mutants probably provoke may promote more efficient product release. The results demonstrate that the mutant hGSTA1-K141H/S142H depicts higher catalytic efficiency and stability than the rest of the mutants studied; the mutation S142H is likely the main contributor. The sites E137 and S142 seem to strongly enhance the catalytic efficiency of the enzyme, and a new more catalytically efficient mutant hGSTA1-1E137H/S142H may be created.

Finally, the motif that was selected seems to play an essential role in catalysis, but also in stability, exhibiting a probable dependent relation with the hydrophobic staple motif (region 153–158). The enhanced stability properties of hGSTA1-K141H/S142H, along with its increased catalytic activity, can make this hGSTA1-1 variant a new potential scaffold for future applications. 

## Figures and Tables

**Table 1:** Steady-state kinetic analysis of hGSTA1-1 and its mutants for the CDNB/GSH substrate system.

Enzyme	K_m_ (mM) (GSH)	* k_cat_^app^ (min^−1^) (GSH)	K_m_ (mM) (CDNB)	k_cat_ (min^−1^) (CDNB)	k_cat_/K_m_ (mM min^−1^) (CDNB)	k_cat_/K_m_ (mM min^−1^) (GSH)
hGSTA1-1	0.28 ± 0.02	607 ± 10	0.27 ± 0.01	625 ± 9	2319 ± 119	2246 ± 193
K141H	0.30 ± 0.02	641 ± 12	0.23 ± 0.02	984 ± 25	4320 ± 484	3301 ± 304
K141H/S142H	0.28 ± 0.02	1371 ± 25	0.16 ± 0.01	1203 ± 23	7547 ± 606	4324 ± 391
K137H	0.29 ± 0.01	1030 ± 8	0.21 ± 0.01	1122 ± 23	5360 ± 365	3876 ± 213
E137H/K141H	0.38 ± 0.03	914 ± 18	0.25 ± 0.02	984 ± 25	3970 ± 417	2610 ± 272

* The values of k_cat_ for GSH of (k_cat_^app^_GSH_) are apparent since it is not always possible to saturate the enzyme with the second substrate.

**Table 2:** The contribution of each mutation in the difference free energy change (ΔG) for stabilization of the transition state (ΔΔG).

Enzyme	ΔΔG (kJ/mol) (CDNB)
hGSTA1-1	N.A.
hGSTA1-K141H	−1.64
hGSTA1-K141H/S142H	−2.97
hGSTA1-E137H	−2.14
hGSTA1-E137H/K141H	−1.38

N.A., Not applicable.

**Table 3:** Half-inactivation temperature (T_1/2_) of hGSTA1-1 and its mutants.

Enzyme	T_1/2_ (°C)
hGSTA1-1	49.6 ± 0.1
hGSTA1-K141H	48.4 ± 0.3
hGSTA1-K141H/S142H	53.4 ± 0.2
hGSTA1-E137H	42.7 ± 0.1
hGSTA1-E137H/K141H	46.4 ± 0.1

**Table 4:** Thermal stability of hGSTA1-1 and its mutants. Reported values represent the average of three replicates.

Enzyme	T_m_ (°C)	ΔuG° (kJ/mol)
hGSTA1-1	55.0 ± 0.1	51.7 ± 0.1
hGSTA1-K141H	54.5 ± 0.1	29.3 ± 0.1
hGSTA1-K141H/S142H	53.2 ± 0.1	52.1 ± 0.1
hGSTA1-E137H/K141H	48.4 ± 0.1	54.3 ± 0.1
hGSTA1-E137H	46.2 ± 0.1	33.7 ± 0.1

ΔuG° is the standard Gibbs free energy of protein unfolding.

**Table 5:** X-ray data collection and refinement statistics.

Data Collection and Processing	hGSTA1-K141H/S142H (High Resolution)	hGSTA1-K141H/S142H (Low Resolution)
Beamline	P13 (EMBL, Hamburg, Germany)	P14 (EMBL, Hamburg, Germany)
Wavelength (Å)	0.9762	0.9762
Resolution (Å)	53.98–1.56 (1.60–1.56)	49.60–1.87 (1.92–1.87)
Space group	*P*2_1_	*P*2_1_2_1_2_1_
Unit cell (Å)		
a, b, c (Å)	54.4, 93.8, 94.1	63.8, 83.2, 96.4
*β* (°)	97.9	90.0
No. of unique reflections	128,429 (7351)	37,747 (917)
Completeness (%)	96.6 (74.9)	94.9 (34.9)
Multiplicity	3.3 (2.6)	4.6 (1.5)
Mosaicity (°)	0.0	0.12
*R*_meas_	0.054 (0.878)	0.048 (1.245)
CC_1/2_	0.99 (0.51)	1.0 (0.51)
Mean (I/σ(I))	13.6 (1.3)	18.6 (0.8)
Wilson B factor (Å^2^)	20.7	37.6
**Refinement**		
No. of reflections used	128,397	37,677
R_cryst_/R_free_	0.163/0.183	0.203/0.236
RMSD in bonds (Å)	0.009	0.009
RMSD in angles (º)	1.20	1.01
No. of non-H atoms (protein/solvent)	6812/957	3361/293
Average B-factor (all/protein/solvent) (Å^2^)	26.6/25.2/36.2	48.0/48.0/48.4
Ramachandran favored/outliers (%)	96.9/0.1	95.4/0.5
Clashscore	3.9	5.4
PDB id	8BHK	8BHE

## Data Availability

Data are contained within the article.

## Supplementary Materials

The following supporting information can be downloaded at: https://www.mdpi.com/article/10.3390/ijms24043700/s1.
