# Supplementary material for: A Key Role in Catalysis and Enzyme Thermostability of a Conserved Helix H5 Motif of Human Glutathione Transferase A1-1
Source: Int J Mol Sci. 2023 Feb 12;24(4):3700. doi: 10.3390/ijms24043700 (PMC9959719; doi:10.3390/ijms24043700)
Supplement: Supplementary file 1 [file ijms-24-03700-s001.zip › ijms-2171725-supplementary.pdf]

SUPPLEMENTARY MATERIAS

Supplementary Table S1. The pairs of mutagenic oligonucleotide primers that were used in the PCR reactions

(K141H)	RPrimer141	5′-GAAAAAGTCTTACATAGCCATGGACA-3′
(K141H)	FPrimer141	5′-TTGTCCATGGCTATGTAAGACTTTTTC-3′
(S142H)	FPrimer142	5´-AAAGTCTTACATCACCATGGACAAGAC-3´
(S142H)	RPrimer142	5´-GTCTTGTCCATGGTGATGTAAGACTTT-3´
(E137H)	F1Primer137	5´-GAAGGAGATACCCTTATGGCAGAGAAGCCCAAGCTCC-3’
(E137H)	F2Primer137	5’-CTTCCCTGCCTTTCATAAAGTCTTACATAGC-3´
(E137H)	R3Primer137	5’-GCTATGTAAGACTTTATGAAAGGCAGGGAAG-3´
(E137H)	R4Primer137	5’-GTGATGATGACCCTTTTAAAACCTGAAAATCTTCCTTGC-3’

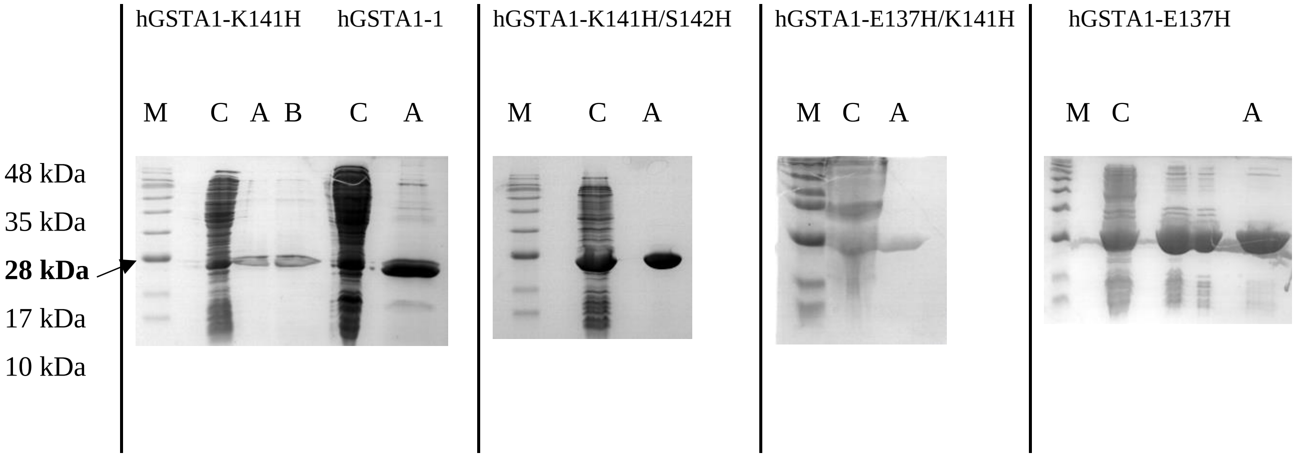

Supplementary Figure S1. Analysis of affinity chromatography purified hGSTA1-1 and its mutants by polyacrylamide gel electrophoresis in the presence of SDS (SDS–PAGE). (M) Pre-stained protein marker (Bluestar Prestained Protein Marker, Nippon Genetics Europe), (C) cell crude protein before purification, (A, B) best fractions of purified enzymes that correspond to the band at ~26 kDa. Each sample contained 50 μg of total protein.

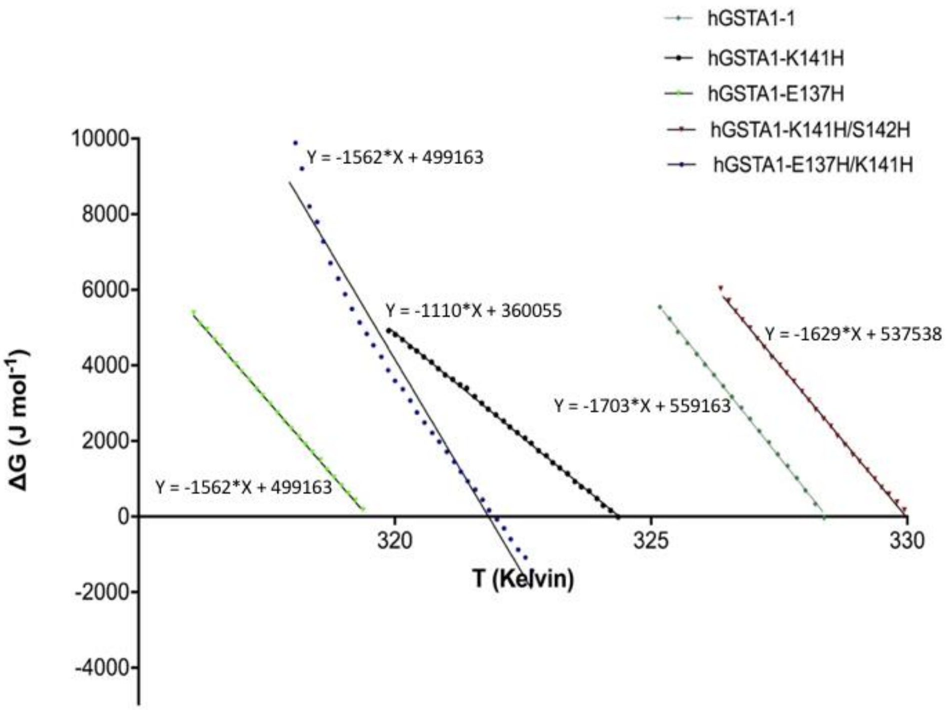

Supplementary Figure S2. Plots of calculated ΔuG values against temperature values corresponding to 10-50% unfolding. The R^2 value for the linear equations is 0.99 for all

the enzymes except of the hGSTA1-E137H/K141H value that is 0.97. Solving for the linear equation of best fit enables ΔuG^o to be calculated.
